# Optimising transcranial direct current stimulation application for the enhancement of exercise performance: a review

**DOI:** 10.3389/fphys.2025.1672603

**Published:** 2025-09-11

**Authors:** Aidan Lewis, Ben Rattray, Andrew Flood

**Affiliations:** ^1^ Discipline of Psychology, Faculty of Health, University of Canberra, Canberra, ACT, Australia; ^2^ Institute of Sports Science, Human Movement Science, University of Bundeswehr, Munich, Neubiberg, Germany; ^3^ Discipline of Exercise Science, Faculty of Health, University of Canberra, Canberra, ACT, Australia; ^4^ University of Canberra Research Institute for Sport and Exercise, University of Canberra, Canberra, ACT, Australia

**Keywords:** tDCS, exercise, optimising, review, enhancement

## Abstract

Transcranial direct current stimulation (tDCS) is a non-invasive brain stimulation technique that has shown potential in enhancing performance across a range of exercise types. However, the variability in its effectiveness suggests that outcomes may be contingent on how stimulation is applied. This review evaluates the current evidence surrounding the optimisation of tDCS for performance enhancement, focusing on individual stimulation parameters; timing, intensity, current density, montage, and electrode configuration, and their interactions. We highlight how modifications in these dose components can produce non-linear and sometimes paradoxical effects on corticospinal excitability, the primary mechanistic rationale proposed for tDCS-related performance gains. Evidence suggests that online vs offline stimulation, session duration, dual-dosing protocols, and extracephalic or high-definition montages can all substantially influence psychophysiological outcomes, though findings remain inconsistent. Through the review, we identify significant gaps in comparative data and cautions against assumptions that increased stimulation intensity or duration equates to improved performance. We critique the reliance on outdated methodologies including the use the 10–20 EEG system, and conclude by providing practical recommendations for future research, calling for systematic investigations of dose interactions, protocol standardisation, and direct comparisons of novel and established tDCS methods. These steps are necessary to utilise tDCS to its full potential in the context of exercise performance.

## 1 Introduction

In the pursuit to enhance athletic performance, a substantial body of research has considered the effects of transcranial direct current stimulation (tDCS) as a performance-enhancing tool. tDCS is a non-invasive neuromodulation technique that involves the administration of a weak direct electrical current to specific brain regions, thereby modulating neuronal activity and related behavioural outcomes (Nitsche and Paulus, 2000). A foundational explanation for its mechanisms of action argued that tDCS induces polarity-specific changes in neuronal membrane excitability; anodal stimulation results in depolarisation, whereas cathodal stimulation causes hyperpolarisation (Nitsche and Paulus, 2000; Nitsche et al., 2003; Priori et al., 1998). In the context of exercise performance, research has demonstrated the performance-enhancing benefits of tDCS across various exercise tasks, including muscular strength tasks (Frazer et al., 2016; Kenville et al., 2020; Vargas et al., 2018) and dynamic, whole-body motor tasks (Codella et al., 2021; Huang et al., 2019; Okano et al., 2015; Park et al., 2019; Vitor-Costa et al., 2015). The reported improvements in exercise performance are often attributed to changes in corticospinal excitability (Angius et al., 2015; Angius et al., 2018; Cogiamanian et al., 2007; Hendy and Kidgell, 2014; Williams et al., 2013), whereby the increase in corticospinal excitability induced by tDCS produces greater motor drive to working muscles and therefore improving the strength and endurance capacities of those working muscles (Cogiamanian et al., 2007; Tanaka et al., 2009). Despite these positive findings, recent systematic reviews highlight the variability in tDCS effects on exercise performance (Chinzara et al., 2022; Shyamali Kaushalya et al., 2022). This heterogeneity suggests that tDCS protocols may require careful optimisation to achieve reliable and consistent performance gains.

A common rationale underpinning the use of tDCS for performance enhancement is the assumption of a linear dose–response relationship: that increasing stimulation intensity or duration will proportionally increase motor cortex excitability and thereby upregulate corticospinal output (Nitsche and Paulus, 2000; Cogiamanian et al., 2007). This upregulation in corticospinal output produces greater motor unit recruitment and motor force production, thereby enhancing performance outcomes (Lattari et al., 2016). Yet, empirical evidence challenges this assumption. For instance, increasing the duration of 1.0 mA anodal tDCS from 13 to 26 min has been shown to reverse expected excitability enhancements, producing inhibitory rather than facilitatory after-effects (Monte-Silva et al., 2013). Similarly, increasing cathodal tDCS intensity from 1 mA to 2 mA unexpectedly resulted in facilitation rather than suppression of excitability (Batsikadze et al., 2013). These non-linear effects underscore the importance of carefully examining the contribution of individual stimulation dose parameters.

The effects of tDCS on both corticospinal excitability and exercise performance likely depend on several key dose parameters, including timing, current density, polarity, intensity, and montage. While these parameters are often examined individually, their effects may not be additive or linear (Hassanzahraee et al., 2020), or consistent across participants (Evans et al., 2020; Perrey, 2024), and the interactions between them could contribute to the variability observed in performance outcomes (Mosayebi et al., 2019). Therefore, this critical review aims to deliver a deeper understanding how each of these different dose components contribute to changes in exercise performance and to better understand the dose-responses to each of those separate elements, and to identify methods to optimise the application of tDCS for exercise performance enhancement. We first explore how each parameter has been manipulated for the optimisation of outcomes in the broader neuromodulation literature—such as in learning or cognitive paradigms—and evaluate whether these approaches have been adopted in exercise contexts. Only after establishing an understanding of each dosage parameter in isolation do we consider how combinations of parameters may interact to influence outcomes. Finally, we offer practical recommendations for refining and standardising tDCS protocols to improve their efficacy and reproducibility in sport and exercise research.

## 2 Optimised applications of tDCS

A wide range of tDCS parameters—such as timing, intensity, current density, polarity, and montage—have been applied in studies of exercise performance, often with inconsistent outcomes. Reviewing how these individual parameters have been used, both within and beyond exercise contexts, can provide important insight into how tDCS protocols might be optimised for more reliable performance enhancement. For the purpose of this review, the exercise contexts that are examined include muscle strength performance, as well as endurance-based tasks, both dynamic and isometric, and excludes motor learning studies.

### 2.1 tDCS timing

Timing is a key consideration in the application of tDCS. Relevant aspects include online versus offline stimulation, stimulation duration, and the use of repeated sessions—all of which are reviewed below in relation to exercise performance.

#### 2.1.1 Online and offline tDCS

An important methodological consideration for using tDCS as an intervention to promote psychophysiological outcomes is the timing of tDCS administration relative to the performance and assessment of that outcome. Online tDCS refers to stimulation during task performance, while offline tDCS is applied either before the task performance and is dependent on tDCS-induced aftereffects (Friehs and Frings, 2019) or after the task performance and is dependant in tDCS-induced consolidation effects (Rumpf et al., 2017). It is believed that online tDCS directly modulates resting membrane potential, upregulating neuronal firing rate and timing, consequently modulating performance outcomes (Farahani et al., 2024; Lafon et al., 2017). In contrast, offline tDCS effects completed before task performance are most likely driven by long-term potentiation mechanisms. In this case, tDCS is presumed to induce plastic changes in the cortex producing prolonged neuronal efficacy, consequently modulating performance outcomes after stimulation cessation (Kuo et al., 2013). Moreover, offline tDCS effects after task performance are most likely driven by consolidative processes; wherein tDCS acts upon the consolidation phases of learning and skill acquisition tasks, with evidence pointing towards a facilitatory effect of tDCS on motor learning (Rumpf et al., 2017).

Some speculate that online tDCS may be more effective than offline tDCS for the modulation of performance outcomes. Bikson and Rahman (Bikson and Rahman, 2013) describe ‘activity-selectivity’, which assumes that tDCS will preferentially modulate specific forms of ongoing activity, like those networks of neurons that are activated during specific tasks. Evidence of this has been reported for cognitive tasks (Conley et al., 2015), motor learning tasks (Besson et al., 2019), and both visual working memory tasks and visuospatial tasks, whereby significant improvements occurred when compared to offline tDCS and continues for up to 24 h (Martin et al., 2014; Sarrias-Arrabal et al., 2023). This effect has also been reported in clinical populations, with Turnbull et al. (2023) reporting a significant amelioration of altered emotion regulation in those who have schizophrenia.

In the exercise performance literature, most studies apply stimulation before the exercise performance assessment (Cogiamanian et al., 2007; Abdelmoula et al., 2016; Angius et al., 2016; Flood et al., 2017a; Kan et al., 2013; Muthalib et al., 2013) rather than during or after. Of these studies, there appears to be mixed effects reported independent of the other dose parameters (i.e., intensity, montage, etc.) (Machado S. et al., 2019). Only two studies have utilised an online application of tDCS during an exercise performance task (Williams et al., 2013; Radel et al., 2017). Radel et al. (2017) applied tDCS at 2 mA for the duration of a 35% isometric grip strength time to exhaustion task and report no significant effect of stimulation on endurance exercise performance when compared to sham stimulation. Williams et al. (2013) applied conventional tDCS over the motor cortex at 1.5 mA for the duration of a 20% elbow flexor time to exhaustion task. The authors report a significant improvement in performance in the time to exhaustion task when stimulation is applied during the task when compared to stimulation delivered before the task. This change in performance occurred without any change in corticospinal excitability (CSE). These findings coincide with recent findings reported by Pillen et al. (2022) who observed no change in CSE during tDCS, pointing to an alternative mechanism by which exercise performance outcomes are modulated by online tDCS. Ultimately, the lack of research makes it difficult to reach conclusions as to whether online or offline tDCS is optimal for exercise performance improvement. Future research should consider providing a direct comparison between the effects of online and offline administration of tDCS on exercise performance.

Despite the importance of the timing of stimulation in relation to the exercise task, the exact timing between stimulation onset or cessation in relation to exercise onset is often underreported. While neurophysiological evidence indicates that offline tDCS-induced increases in CSE peak shortly after stimulation (∼15–30 min) (Santarnecchi et al., 2014), and that extended durations or delays can even reverse these effects (Vaseghi et al., 2015), most exercise studies fail to explicitly report or justify their chosen time interval (Huang et al., 2019; Cogiamanian et al., 2007; da Silva Machado et al., 2021; Holgado et al., 2019a; Lattari et al., 2020). Moreover, the majority of investigations initiate exercise immediately post-stimulation without experimentally examining whether this practice indeed maximises performance enhancement (da Silva Machado et al., 2021; Angius et al., 2019; Baldari et al., 2018; Byrne and Flood, 2019; Etemadi et al., 2023). And though the reversal of effects on CSE isn’t as relevant for online tDCS as offline tDCS (as the enhancement of effects is via different mechanisms), the timing of the application of online tDCS in relationship to the onset of exercise is still of importance and is still often omitted from the literature. For example, Santarnecchi et al. (2014) report a 7% difference in the size of MEPs during the time course of an application of online tDCS over 15 min, with the largest increase in MEPs being 2.5 min after stimulation onset, indicating optimal timing parameters for online tDCS for the enhancement of cortical excitability.

In the two studies examining the online application of tDCS, both fail to provide a sound rationale for when the stimulation is applied in relation to the exercise task. Radel et al. (2017) report that after a 2 min rest period, tDCS began for 10 min before the onset of the exercise task. However, the rationale for this decision is unclear in light of the findings of Santarnecchi et al. (2014) outlined above. Similarly, in a study conducted by Williams et al. (2013), the online application of tDCS was initiated 1.5 min into a fatiguing isometric elbow contraction and lasts the entire duration of the contraction, with the authors justifying this approach by specifying that anodal tDCS delivered for 7 min has been shown to be sufficient to modify cortical excitability with changes that persist for 5–10 min after turning off the current. Though justified, the rationale is unclear considering the enduring effects of tDCS has little relevance to an online application of tDCS (Santarnecchi et al., 2014). Together, the omission of statements clarifying stimulation cessation relative to exercise onset, as well as providing weak justification for the timing when it is specified, hinders the interpretation and reproducibility of findings, as it remains unclear whether exercise tasks were performed within optimal neuromodulatory windows. Future studies should explicitly document the precise timing between stimulation end and exercise onset in the context of offline tDCS. Regarding online tDCS, future studies should base their justifications from optimal stimulation windows during stimulation. The justification for the relationship between both offline and online tDCS to the onset of exercise performance should be based on established neurophysiological timelines, and systematically explore varying timing conditions. Doing so would enhance the methodological rigour and consistency in the field.

#### 2.1.2 Stimulation duration

The duration of the stimulation protocol is another component of tDCS timing that may be optimised to produce greater effects on exercise performance. The physiological rationale for this possibility is imbedded in the assumption that the effects of tDCS on CSE increase linearly with longer stimulation durations (Nitsche and Paulus, 2000); the proposed mechanism through which tDCS is thought to increase exercise performance (Angius et al., 2015; Cogiamanian et al., 2007; Hendy and Kidgell, 2014; Williams et al., 2013). However, the notion of longer stimulation periods leading to longer lasting effects is not necessarily supported by recent data. Instead, a non-linear response has been observed, with some reporting reductions in CSE as stimulation periods are increased (Mosayebi et al., 2019). Indeed, in some cases, longer stimulation duration has been shown to reverse the effects of anodal tDCS from expected increased excitability to decreased excitability (Monte-Silva et al., 2013; Hassanzahraee et al., 2020). The results of these studies show that increasing the duration of tDCS does not necessarily enhance its effects on CSE and may even reverse the direction of effects.

While the effect of stimulation duration on CSE has been examined, little research has considered whether the performance-enhancing effect of tDCS depends on stimulation duration. In the exercise performance literature, the majority of applications of tDCS range from 10 min to 20 min. For example, Cogiamanian et al. (2007) report significant improvements in elbow flexor endurance time following 10 min of tDCS, though others have reported no performance gains following stimulation of the same duration (Kan et al., 2013). In studies applying 20 min of stimulation, there appears to be similar inconsistencies in findings with Frazer et al. (2016) reporting increased performance and Angius et al. (2018) reporting no change in performance following tDCS with identical stimulation parameters. Several meta-analyses have reported on the duration of stimulation with similar heterogeneity (Holgado et al., 2019b). For example, Chinzara et al. (2022) in their recent meta-analysis report no evidence that stimulation duration moderates performance-enhancing effects. It may therefore be necessary to conduct repeated measures designs for performance outcomes, titrating the timing of stimulation, similar to what has been done in the studies investigation the effects of stimulation on CSE, to discover the optimal stimulation parameter for exercise performance enhancement.

#### 2.1.3 Single session multiple dose tDCS

A third factor in the timing of stimulation that may lead to the optimisation of tDCS application is the administration of multiple tDCS doses within a small timeframe (∼ 1 h). It has recently been speculated that by leveraging underlying metaplastic mechanisms, the application of multiple tDCS doses in succession would promote its effects greater than a single dose (Hurley and Machado, 2017). Metaplasticity is a higher-order form of synaptic plasticity that is tightly affected by the history of synaptic activity (Abraham, 2008), whereby high previous neuronal activity would likely lead to long-term depression, whereas low previous neuronal activity would likely lead to long-term potentiation (Bienenstock et al., 1982; Karabanov et al., 2015; Müller-Dahlhaus et al., 2015). Differential and improved effects on CSE can be obtained when applying short- or long-lasting intervals between doses (Fricke et al., 2010). For example, Monte-Silva et al. (2013) applied a single dose of tDCS for 26 min, and two doses at 13 min with a 3 min interval. The authors report that the single dose at 26 min induces a significant reduction in motor evoked potentials (MEPs), but the split dose produced the opposite effect with a significant enhancement in MEPs. A meta-analysis investigating this double dose effect across all non-invasive brain stimulation techniques report a consistent capacity of an initial dose to prime the subsequent dose, thereby producing larger effects on the target outcome (Cosentino et al., 2012; Hassanzahraee et al., 2018). Indeed, these effects seem to extend beyond the physiological alteration of changes in CSE, with some studies showing improved effects on memory (Carvalho et al., 2015) skill acquisition (Fujiyama et al., 2017), and visuo-motor learning (Besson et al., 2020) after the application of dual dosage tDCS.

While multiple doses of tDCS appear to leverage metaplastic mechanisms and enhance effects on CSE, few studies have examined the effects of this optimised method of tDCS administration on exercise performance duration. One such method of applying multiple doses of tDCS is called cathodal preconditioning, where an initial inhibitory dose of cathodal tDCS is applied prior to an excitatory dose of anodal tDCS (Pourmajidian et al., 2020). Recent findings have shown that MEPs during a fatiguing grip strength task are elevated after the application of this cathodal preconditioning technique, but there is no assessment of exercise performance endurance itself (Boda et al., 2024; Xian et al., 2023). Further, while not assessing changes in exercise performance directly, one study examined the effect of dual dosage tDCS combined with a cycling task on MEPs (Pourmajidian et al., 2020). In this study, participants received either a preconditioning or sham dose of cathodal tDCS at 2 mA for 10 min, before a combined anodal tDCS and cycling intervention. Here, participants cycled for 10 min with a power output of 120% of their body weight at a cadence of 80 RPM while receiving anodal tDCS. The authors report that cathodal preconditioning boosted CSE after the combined intervention greater than the sham cathodal preconditioning dose. While a cycling task was included in the study, no performance outcomes were reported, limiting our ability to draw conclusions as to the effect of the novel tDCS application on performance. One other study has since utilised this approach for the enhancement of exercise performance. Lewis et al. (2025) examined the effects of cathodal preconditioning on CSE and isometric grip strength and endurance. The authors report that cathodal preconditioning did not significantly improve endurance performance on a grip strength task, and there was no significant change in CSE before and after the stimulation dose. Overall, dual dose tDCS appears to increase CSE, but only when paired with cycling or another dynamic intervention. However, changes in CSE do not seem to be associated with improvements in exercise performance.

#### 2.1.4 Multiple tDCS sessions

It has been posited that the effects achieved from single sessions of tDCS are relatively quite small (Hamilton et al., 2019). Therefore, to optimise its application, repeated stimulated sessions of tDCS over multiple days and weeks are often utilised to produce larger, enduring, and clinically relevant impacts (Ferrucci et al., 2014; Martin et al., 2013). Repeated sessions have been shown to amplify and prolong tDCS-induced plasticity (Goldsworthy et al., 2015), and cumulative increases in cortical excitability emerge when sessions are repeated (Alonzo et al., 2012). When applied clinically, the application of tDCS over repeated sessions has found a significant reduction in depressive scores (Couture et al., 2025), an amelioration of chronic pain symptoms in fibromyalgia (Villamar et al., 2013), as well as the amelioration of tinnitus symptoms (Faber et al., 2012). One study, however, has reported that changes in motor learning outcomes after tDCS over three consecutive days is not significantly different to sham tDCS (Besson et al., 2020). Collectively, these findings suggest that delivering tDCS across several sessions may be a promising strategy for maximising behavioural outcomes.

To date, however, only one study has investigated whether multi-session tDCS can enhance exercise performance. Despite a robust mechanistic rationale—the cumulative modulation of neural excitability as outlined above—this dosing approach has only been adopted in the sport and exercise domain once. Zhang et al. (2025) applied stimulation at 1 mA, 2 mA, and 3 mA over 10 sessions and found that both 2 and 3 mA intensities significantly improved both a countermovement jump and isokinetic maximal peak torque production. The authors report no significant change in performance outcomes after a single session, and no change in performance outcomes after 1 mA stimulation (Zhang et al., 2025). Beyond this study in healthy participants, multi-session tDCS have been used to support motor recovery in multiple sclerosis (Ferrucci et al., 2014; Mattioli et al., 2015), stroke (Tedesco Triccas et al., 2016), and Parkinson’s disease (Benninger et al., 2010). For example, in stroke rehabilitation, repeated tDCS has improved functional task performance and muscle strength (Fusco et al., 2013), providing further theoretical support for similar gains in healthy individuals for motor performance enhancements (Sanes and Donoghue, 2000). Together, these findings indicate that performance improvements are not only feasible but may parallel those observed in clinical populations, where consecutive sessions are often required to achieve meaningful changes. This raises the possibility that multi-session tDCS could be more effective than a single application of tDCS in enhancing exercise performance. Future research is therefore needed to continue to test this hypothesis and to develop a clear understanding if and how multi-session tDCS should be administered and optimised to maximise exercise performance outcomes.

### 2.2 tDCS intensity

The intensity of electrical current delivered to the brain via tDCS is another opportunity for the optimisation of the technique for improvements in exercise performance. Current intensity is the total amount of current that is delivered to the electrodes via the tDCS device, and typically ranges from 0.5 to 2 mA (Brunoni et al., 2012). It is generally accepted that the current flow intensity in the brain will increase linearly with applied current (Bikson et al., 2015). However, individual anatomical differences may produce complex and variable electric field distributions across the brain, which may therefore lead to inter-individual variations in CSE (Bestmann and Ward, 2017; Wang et al., 2022). Nonetheless, the focus below is on the impact of applied current intensity while noting how other factors (i.e., variability in anatomical structures) may influence the current-intensity dose.

Early research by Nitsche and Paulus (2000) suggested that greater stimulation intensity would result in increasing neuronal excitability changes. The authors titrated tDCS intensity from 0.2 mA to 1 mA in 0.2 mA steps and reported a linear increase in CSE. However, more recent evidence suggests that this linear increase cannot be assumed (Batsikadze et al., 2013; Bastani and Jaberzadeh, 2013a; Esmaeilpour et al., 2018; Kidgell et al., 2013). For example, it has been reported that 1 mA tDCS induced an expected decrease with the cathode over the motor cortex, but this effect was reversed at 2 mA (Batsikadze et al., 2013). One titration study of a wide range of current intensities (i.e., 0.3, 0.7, 1.4 and 2 mA) exhibited greater CSE facilitation with 2 mA stimulation than 1.4 mA (Bastani and Jaberzadeh, 2013a). However, the same study also illustrated larger effects on CSE with 0.3 mA than with 0.7 mA, suggesting a partial non-linear relationship between current intensity and excitability outcomes (Bastani and Jaberzadeh, 2013a). It has been speculated that the origin of this non-linearity relates to the flux of sodium in post-synaptic neurons, whereby low postsynaptic calcium enhancement causes long-term depression (LTD), and large calcium increases result in long-term potentiation (LTP) (Nitsche et al., 2003; Kronberg et al., 2017; Malenka and Bear, 2004). Alternatively, the non-linear effects may be determined by the axonal orientation relative to the electric field vector, from which it follows that tDCS-induced homogenous electric fields do not uniformly modulate all neurons in the stimulated area (Kabakov et al., 2012). It may be that the changing of current intensity consequently alters the dendritic depolarization to a level which has an impact on whole neuronal excitability or resulted in polarization of structures with different neuronal orientation, therefore producing differences in plasticity (Batsikadze et al., 2013). Ultimately, the unclear origin of non-linear changes impact on the proposed exercise-performance enhancing effects of tDCS, which are thought to be mediated by a change in CSE.

In studies examining the exercise performance enhancing effects of tDCS, varying stimulation intensities have been used. The most common current intensities employed in the literature are 1.5 mA (Cogiamanian et al., 2007; Williams et al., 2013; Abdelmoula et al., 2016; Hikosaka and Aramaki, 2021) and 2 mA (Hendy and Kidgell, 2014; Kan et al., 2013; Muthalib et al., 2013; Radel et al., 2017). Cogiamanian et al. (2007) applied tDCS at 1.5 mA for 10 min and found a significant increase in endurance performance relative to cathodal and control conditions. However, following the work of Cogiamanian et al. (2007), Muthalib et al. (2013) reported no significant change in the time to exhaustion (TTE) of the left elbow flexors following anodal tDCS over the primary motor cortex at an increased stimulation intensity (2 mA). This different finding may be due to the increased stimulation intensity, as recent research by Agboada et al. (2020) has indicated that even small changes in stimulation intensity (∼0.5 mA) can result in fluctuations between long-term potentiation and long-term depression. In a systematic review of the exercise performance literature, Holgado et al. (2019b) reported no significant effect of stimulation intensity on exercise performance outcomes. Indeed, in a recent study examining the difference between 1 and 2 mA intensities on exercise performance, Wrightson et al. (2020) reported that neither one nor 2 mA tDCS improved exercise performance when compared with sham stimulation. These inconsistencies limit possible recommendations for the optimal stimulation intensity to produce improvements in exercise performance. Further research comparing the performance enhancing effects of tDCS across multiple stimulation intensities is therefore warranted.

Updated safety limits proposed in 2018 provided opportunities to examine stimulation intensities exceeding 2 mA (Nitsche and Bikson, 2017). Some tDCS studies have reported stronger effects with higher current doses without jeopardizing safety and worsening tolerability (Jamil et al., 2020; Trapp et al., 2019). Indeed, several others have reported improvements in motor learning (Hsu et al., 2023) and working memory (Roncero et al., 2021) after 4 mA tDCS. These changes appear to be related to changes in CSE, with research reporting significantly enhanced CSE after 3 mA tDCS when compared to regular (1–2 mA) tDCS (Mosayebi et al., 2019). Workman et al. (2020) investigated the effects of 4 mA anodal tDCS over the primary motor cortex on performance fatigability and EMG activity of the leg muscles during a maximal isokinetic task in healthy young adults. The authors report that 4 mA tDCS did not affect torque production and EMG activity compared to sham. Zhang et al. (2025) directly compared the effects of 1 mA, 2 mA, and 3 mA on countermovement jump height and reported no significant changes in height from pre-to post-stimulation. Considering most studies in the exercise performance literature investigate the effects of tDCS on isometric tasks (Cogiamanian et al., 2007; Abdelmoula et al., 2016; Kan et al., 2013; Lampropoulou and Nowicky, 2013) or dynamic exercise tasks (Huang et al., 2019; Vitor-Costa et al., 2015; Angius et al., 2018; Sasada et al., 2020), it is unclear whether this negative finding can be attributed to the high intensity stimulation, or whether the isokinetic task or countermovement jump task, which are rarely used, may have influenced the negative finding.

### 2.3 tDCS montages

Electrode montage is the another key consideration in the application of tDCS. Relevant aspects include the targeted brain regions of interest and optimised methods for targeting those regions, the location of the reference (cathodal) electrode, and high-definition tDCS (HD-tDCS) protocols - all of which are reviewed below in relation to exercise performance.

#### 2.3.1 Targeted brain regions

In research exploring the performance-enhancing potential of tDCS, several brain regions have been targeted, each chosen based on its hypothesised role in facilitating exercise performance. The motor cortex remains the most frequently stimulated region due to its fundamental role in planning, coordinating, and executing voluntary movement (Sanes and Donoghue, 2000). Several studies have reported improvements in exercise performance following M1 stimulation coinciding with an increase in CSE (Cogiamanian et al., 2007; Williams et al., 2013). However, this relationship is not always consistent (Abdelmoula et al., 2016), and many studies have assumed changes in CSE without directly measuring it (Park et al., 2019; Vitor-Costa et al., 2015; Sasada et al., 2020). Such assumptions are problematic because other mechanisms, such as the reduction of perceived pain, may underlie observed performance improvements following M1 stimulation (Flood et al., 2016).

Beyond the motor cortex, the dorsolateral prefrontal cortex (DLPFC) has also been investigated as a promising stimulation target due to its critical role in inhibitory control and the regulation of sustained cognitive effort during exercise (Hagger et al., 2010). Indeed, tDCS applied over the DLPFC appears particularly beneficial for exercise tasks that induce significant mental fatigue, such as prolonged cycling or resistance tasks requiring sustained effort (Lattari et al., 2016; Etemadi et al., 2023). Similarly, anodal tDCS over the temporal cortex has been explored based on its involvement in processing sensory information relevant to exercise (Hilty et al., 2011), modulating autonomic nervous system responses (Oppenheimer et al., 1992), reducing perceived effort and fatigue (Montenegro et al., 2011), and contributing to motor control perception (Pelphrey et al., 2005). Temporal cortex stimulation has been shown to improve cycling performance through reductions in heart rate and perceived exertion (Okano et al., 2015), though these effects have not been consistently observed across other tasks, such as isokinetic knee extensions (Ciccone et al., 2019). Lastly, stimulation over the cerebellum, despite being less extensively studied, has shown promise due to its central role in balance and coordination (Morton and Bastian, 2004). For example, cerebellar tDCS appears more effective than M1 stimulation for balance-oriented exercises such as barbell squats (Kenville et al., 2020).

Collectively, these findings emphasise the importance of aligning the brain region targeted by tDCS with the physiological and psychological demands of the exercise task. Yet, achieving such alignment is complicated by considerable heterogeneity in how performance outcomes are defined and measured. For instance, endurance performance has variously been evaluated through time-to-exhaustion tasks, time-trial completions, total work outputs, or subjective ratings of exertion (Cogiamanian et al., 2007; Angius et al., 2016; Radel et al., 2017; Lewis et al., 2025; Lewis et al., 2023). Strength performance measures similarly vary, including maximal voluntary contractions, one-repetition maximum lifts, repetitions to failure, or torque and power output (Vargas et al., 2018; Abdelmoula et al., 2016; Kan et al., 2013; Lattari et al., 2020; Workman et al., 2020; Lampropoulou and Nowicky, 2013). Importantly, these outcome metrics are not interchangeable and appear to respond differently to tDCS (Holgado et al., 2024). Indeed, a meta-analysis identified significant improvements in endurance when defined by sustaining a submaximal contraction, yet no clear benefits when endurance was quantified as total work completed (Hilty et al., 2011). Additionally, strength-related outcomes have generally demonstrated more consistent benefits (∼67% improvement rates) compared to endurance measures (∼50%) following anodal tDCS (Machado DGdS. et al., 2019).

Such discrepancies might arise because exercise performance itself is multifaceted, demanding diverse cognitive and physiological regulation. Different exercise tasks, whether strength or endurance, complex or simple movements, involving large or small muscle groups, focusing on skill-learning or maximal effort, all engage distinct neural pathways and psychophysiological processes (Steinberg et al., 2019; Qi et al., 2024). Thus, optimal tDCS outcomes depend critically on selecting brain targets that directly modulate these specific task-relevant mechanisms. Whole-body endurance tasks, for example, involve cardiovascular responses, pacing strategies, motivation, and sensory integration—complex factors that tDCS may only selectively influence (Jaberzadeh and Zoghi, 2022). In contrast, strength tasks or localised endurance assessments more directly reflect corticospinal drive to working muscles, providing clearer pathways for tDCS-induced improvements (Angius et al., 2017). Given this complexity, researchers must adopt clear, hypothesis-driven rationales for how stimulation is expected to influence specific aspects of exercise performance, recognising that tDCS can have either facilitating or inhibiting effects depending on task–brain alignment. Furthermore, beyond assessing corticospinal excitability, manipulation checks examining additional physiological and psycho-cognitive factors—such as motivation, perceived exertion, pain perception, or sensory processing—should be routinely incorporated.

Currently, very few studies have directly compared the efficacy of stimulating different brain regions for exercise enhancement, limiting the ability to draw definitive conclusions. Among the available studies, Etemadi et al. (2023) reported that DLPFC stimulation was more effective than M1 for submaximal endurance cycling. In contrast, Isis et al. (2023) found no significant performance enhancement from stimulating either the M1 or temporal cortex during maximal incremental cycling tasks, despite increases in cortical excitability following M1 stimulation. Anoushiravani et al. (2023) reported that premotor cortex stimulation led to greater enhancements in strength, power, speed, and coordination among professional gymnasts compared to cerebellar and sham stimulations. These limited comparative findings highlight the necessity of clearly aligning tDCS target sites with specific exercise tasks and mechanisms. Therefore, future research should prioritise direct comparisons across multiple stimulation targets and quantify underlying physiological or psychological mechanisms rather than assume their involvement. Such an approach would clarify the optimal pairing of brain regions and stimulation parameters to maximise tDCS-induced exercise performance enhancements.

##### 2.3.1.1 Methods for targeting brain regions

To date, most clinical and experimental studies that use tDCS have adopted the International 10–20 EEG system to guide electrode placement (Lioumis and Rosanova, 2022). In this approach, an EEG cap is positioned according to easily palpated cranial landmarks such as the nasion, inion, and pre-auricular points, and the cortical target is inferred from its proportional distance to those landmarks (Herwig et al., 2003). Although the 10–20 system partially compensates for inter-individual differences in head size, it ignores variations in head shape as well as the gyral and sulcal morphology of the underlying cortex (Uylings et al., 2005). Recent work has therefore focused on more precise, participant-specific montage design. First among these innovations is MRI-guided neuronavigation, which uses each participant’s structural MRI together with a frameless navigation system to position electrodes directly over the intended cortical region (Lioumis and Rosanova, 2022; Souza et al., 2018). Compared with scalp-based heuristics, neuronavigation substantially reduces spatial error and improves confidence that the electrodes truly overlie the region of interest, therefore aiding in the control of interparticipant variable (Julkunen et al., 2009; Lefaucheur, 2010; Peleman et al., 2010). When applied clinically, empirical evidence indicates that personalised, MRI-guided montages yield larger and more reliable behavioural effects than conventional 10–20 placements (Tsukuda et al., 2025).

Electric-field-guided computational modelling provides a complementary route to precision tDCS. In contrast to MRI-guided neuronavigation—which uses anatomical MRI landmarks to place electrodes over a visible cortical target—electric-field modelling begins by building a subject-specific finite-element head model from the same structural MRI data. The model incorporates conductivity boundaries for skin, skull, cerebrospinal fluid, and any lesion tissue, allowing simulation of how a given montage shapes the intracranial electric field (Yoon et al., 2024). Optimisation algorithms then adjust electrode size, location, and current intensity to maximise field strength within a predefined cortical target while constraining off-target spread (Datta et al., 2011; Parazzini et al., 2017). In silico studies illustrate the gain in focal dose: in stroke, a personalised montage generated by e-field optimisation delivered substantially higher current to the lesioned motor cortex than the canonical placement achieved by simply repositioning the pads (van der Cruijsen et al., 2023). By tailoring stimulation to each person’s head anatomy, electric-field modelling is emerging as a key strategy for enhancing the focality and therapeutic efficacy of tDCS.

In contrast to the advanced targeting strategies outlined above, most tDCS investigating exercise performance continue to rely on electrode placement based on the 10–20 EEG system. Typically, researchers position the anode on standard sensorimotor or prefrontal sites—most often C3 (Vitor-Costa et al., 2015; Flood et al., 2016), Cz (Etemadi et al., 2023; Isis et al., 2023) or F3 (Etemadi et al., 2023; Vieira et al., 2020)—while positioning the cathode over the contralateral supra-orbital region. More precise methods, including MRI-guided neuronavigation and individualised electric-field modelling, remain rare within this literature (Vieira et al., 2020). While the standard 10–20 EEG approach offers practicality, particularly given the significant expense and time demands associated with MRI-based methods, this practicality comes at the cost of spatial accuracy (Brunoni et al., 2012). Computational modelling and empirical evidence clearly demonstrate that electrode-placement errors of even a few centimetres can substantially alter intracranial electric-field distribution, potentially diminishing or redirecting the intended effects of stimulation (Indahlastari et al., 2023; Woods et al., 2015). Therefore, despite practical challenges, it is advisable to incorporate advanced targeting techniques such as MRI guidance and electric-field modelling wherever feasible. Without such methods, caution is warranted when interpreting tDCS outcomes, as variability in electrode placement inevitably contributes to inconsistencies in stimulation dose and resultant effects across both participants and studies.

#### 2.3.2 HD-tDCS

High-definition tDCS (HD-tDCS) was developed on the basis of computational modelling studies showing enhanced focality of current flow when using the multi-electrode array montage (Kuo et al., 2013). HD-tDCS montages use smaller electrodes than conventional tDCS allowing the electric current to be delivered with increased density and focality. The most used HD-tDCS montage is the 4 × 1 ring configuration, which consists of one anodal electrode surrounded by four cathodal electrodes. In this way, the delivered electric current is constrained and localised within the ring of return electrodes (Edwards et al., 2013). Finite element method (FEM) models predict a different strength and distribution of electric field induced by conventional and HD-tDCS (Datta et al., 2009). In particular, electrode placements of HD-tDCS contribute to reduce the uncontrolled diffusion of tDCS-induced electric fields, thereby improving the spatial precision with which the electrical current can target specific cortical regions (Kuo et al., 2013; Hill et al., 2018). Indeed, this increase in spatial precision has coincided with significant increases in CSE after stimulation when compared to a conventional tDCS montage (Kuo et al., 2013). HD-tDCS targets the brain region with greater precision and is argued to produce greater changes in excitability and consequent changes in target tasks, with evidence showing greater improvements in cognitive performance following HD-tDCS compared to conventional tDCS (Parlikar et al., 2021).

Whilst HD-tDCS has been proposed to facilitate more focal enhancement of CSE, these alterations have not yet been shown to manifest in improvements in endurance performance (Machado S. et al., 2019; Radel et al., 2017; Flood et al., 2016). The application of HD-tDCS has been shown to improve excitability of the motor cortex and prefrontal cortex, these results were accompanied by no significant differences in TTE in a sustained isometric contraction of the elbow flexors, fatigue indices and perceived exertion (Radel et al., 2017). Likewise, Flood et al. (Flood et al., 2017b) observed that improvements in experimental pain threshold were not accompanied by improvements in maximal force or TTE of the knee extensors, following the application of HD-tDCS to the motor cortex. These null findings have been reproduced in a randomised controlled trial, whereby HD-tDCS over the motor cortex produced no significant difference in a cycling TTE task, perceived exertion, or heart rate when compared to sham stimulation (da Silva Machado et al., 2021). The lack of performance-enhancement suggests that while HD-tDCS may influence cortical excitability, it may not directly translate to enhanced outcomes in endurance exercise tasks.

#### 2.3.3 Extracephalic tDCS

Common tDCS montages apply both the anode and cathode over the scalp, with the anode placed over the target region and cathode place over the contralateral supraorbital area, as this seemed to induce the most consistent and favourable injection of current into the brain (Nitsche and Paulus, 2000). However, an extracephalic montage, involving the placement of the cathode on either the ipsilateral or contralateral shoulder, may present as an optimised application of tDCS for several reasons. First, as previously mentioned, the polarity of the current (anodal versus cathodal) has a direct influence on the modulation or suppression of CSE (Nitsche and Paulus, 2000). It may therefore be beneficial to remove the proposed inhibitory effect of the cathode over the frontal regions when located over the supraorbital area by changing its location from the scalp to the shoulder (Noetscher et al., 2014). Second, it has been suggested that moving the cathode from the scalp induces a greater concentration of electric currents (Noetscher et al., 2014; Mendonca et al., 2016). When compared to cephalic configurations, extracephalic montages induce a significant amount of current directly underneath the active electrode, rather than between the electrodes (Noetscher et al., 2014; Mendonca et al., 2016). Lastly, it is suggested that extracephalic tDCS may produce electric fields in deeper brain structures (e.g., cerebellum, thalamus and striatum midbrain, pons and medulla) compared to cephalic montages (Parazzini et al., 2013).

The application of an extracephalic tDCS montage has been adopted in the exercise performance literature. Angius et al. (2016) compared the effects of different stimulation montages and reported that extracephalic anodal stimulation over the motor cortex was more efficient for improving performance compared to cephalic montage. Later, Angius et al. (2018) investigated if an extracephalic montage could be improved upon by applying the same montage concomitantly on the contralateral side–that is, applying two anodes over the two hemispheric motor cortices, and cathodes over the ipsilateral shoulders. The authors report a significant improvement in endurance cycling performance but only compared to a concomitant extracephalic cathodal montage and sham stimulation without comparing to a single administration of cephalic or extracephalic tDCS. Using the same concomitant extracephalic montage, Zhiqiang et al. (2023) report a significant improvement in jump height when compared to sham stimulation. A similar limitation occurs here, where the authors fail to compare the dual extracephalic montage to a single extracephalic or cephalic montage.

The conclusions that can be drawn for existing research examining the performance enhancing potential of extracephalic montages are limited for several reasons. Firstly, as highlighted above, it is speculated that performance improvements may be related to increased current density at focal target regions of the brain (Noetscher et al., 2014); however, no studies examining exercise performance after extracephalic tDCS have assessed or quantified changes in CSE. Therefore, it is unclear whether the proposed increase in current density has occurred. Second, the assumption that removing the cathode from the frontal regions will always eliminate inhibitory effects oversimplifies the widely-observed non-linear effects of both anodal and cathodal tDCS on physiological outcomes (Batsikadze et al., 2013). Third, other methods of increasing current density and focality appears to not change exercise performance outcomes, as evidenced by null findings when applying HD-tDCS (Machado S. et al., 2019; Radel et al., 2017; Flood et al., 2016). These findings question the suggestion that greater focality from extracephalic stimulation will result in greater performance gains. Lastly, claims of deeper brain structure stimulation lack robust empirical evidence and often rely on theoretical models and have not been confirmed through conclusive experimental data. Therefore, while extracephalic montages may present some theoretical advantages, their purported benefits require more rigorous validation through empirical research.

### 2.4 Electrode size

The size of the electrodes used in tDCS is a critical determinant of current density, a key factor influencing stimulation outcomes. Current density, expressed in mA/cm^2^, is calculated as the ratio of current intensity (mA) to electrode size (cm^2^) (Ho et al., 2016). Adjusting electrode size then is critical in the modulation of the physiological and behavioural effects of tDCS, for adjusting the size of the electrode can alter both the spatial focality of the electric field and the current density at cortical regions of interest (Bastani and Jaberzadeh, 2013b; Mikkonen et al., 2020). For example, Nitsche et al. (2007) found that reducing the size of the anode electrode increased the focality of motor cortex excitability changes, while increasing the size of the cathode electrode maintained this increase in motor cortex excitability effects, but for an extended duration when compared to decreasing anodal electrode size. This strategy highlights the potential of electrode size manipulation to improve the precision of tDCS. However, research has also identified limitations associated with large electrodes. Mondino et al. (2016) observed that 10 × 10 cm electrodes produced minimal effects on cognitions possibly due to excessive current dispersion.

Beyond targeting precision, electrode size has a significant role in modulating the current density at cortical targets (Ho et al., 2016). For example, Bastani and Jaberzadeh (2013a) found that anodal tDCS delivered at a lower current density of 0.013 mA/cm^2^ (0.3 mA, 24 cm^2^) was more effective at inducing increases in excitability compared to a higher and more typically used current density of 0.029 mA/cm^2^ (0.7 mA, 24 cm^2^), suggesting a non-linear effect of stimulation. Conversely, other work has found no significant differences in excitability changes between current intensities of 0.8–1.2 mA (0.032–0.048 mA/cm^2^, 25 cm^2^ electrodes) (Kidgell et al., 2013). Delineating the effects of electrode size and current density, Bastani and Jaberzadeh (2013b) applied a-tDCS using three active electrode sizes (12, 24, and 35 cm^2^) at a constant current density of 0.029 mA/cm^2^, with a fixed reference electrode size of 35 cm^2^. They observed that smaller electrodes produced a more focal electric field and potentially more effective, localized neural modulation compared to larger electrodes. Collectively, these findings suggest that electric field focality, rather than current density alone, may be the more critical parameter for optimising stimulation effects.

In exercise performance studies, Kenville et al. (2020) utilised a large cathode electrode (100 cm^2^, current density: 0.020 mA/cm^2^) to increase the focality of the injected current into the cerebellar using a conventional anode electrode size (35 cm^2^, current density: 0.057 mA/cm^2^). This electrode setup enabled targeted stimulation of the cerebellum and produced significant performance-enhancing effects on an isometric barbell squat. Regarding the influence of electrode size on current density in the context of exercise performance, a recent meta-analysis investigating the acute effects of tDCS on athletic performance report specifically that current density could only explain 1.25% of the heterogeneity of the changes in observed performance outcomes, a percentage score that was insignificant (p = 0.262). Together, these findings emphasise the importance of electrode size in modulating electric field focality and the need to better understand the influence of current density to maximise the efficacy of tDCS, particularly in applications like exercise performance.

### 2.5 Interaction between dose parameters

Optimising tDCS protocols require considering not only individual dose components such as current intensity, current density, and stimulation duration, but also the complex interactions between these variables. The relationship between intensity and duration is particularly crucial, as tDCS effects often demonstrate non-linear patterns rather than straightforward dose-response relationships. For example, Bastani and Jaberzadeh (2013a) reported increased CSE when intensity was raised from 1.4 mA to 2 mA. Conversely, Mosayebi et al. (2019) found that 2 mA stimulation for 20 min induced LTP-like plasticity, while shorter or longer durations at lower intensities paradoxically resulted in LTD-like effects. These observations highlight that interactions between intensity and duration are not merely additive but may shift the directionality and magnitude of neuroplastic changes. The underlying reason for this non-linearity likely involves calcium dynamics within neuronal populations, which are critical in shaping neuroplastic outcomes (Mosayebi-Samani et al., 2020). Specifically, the amount of calcium influx into neurons differ depending on the precise combination of stimulation intensity and duration. At certain thresholds, calcium levels may support LTP induction, whereas other combinations might preferentially trigger calcium-dependent mechanisms of LTD.

In the exercise performance literature, similar complexity is observed. Abdelmoula et al. (2016) reported significant improvements in isometric TTE with 1.5 mA stimulation for 10 min, though studies using 2 mA for the same duration reported no significant effects (Kan et al., 2013; Muthalib et al., 2013). However, extending the stimulation duration to 20 min at 2 mA resulted in significant performance enhancements (Workman et al., 2020), underscoring the importance of duration in modulating tDCS outcomes. Interactions between intensity and cortical target region further complicate dose-response relationships. Abdelmoula et al. (2016) showed performance benefits with 1.5 mA applied over the motor cortex, whereas stimulation at lower intensity (1 mA) over the DLPFC was ineffective (Angius et al., 2018). At higher intensity (2 mA), findings diverge based on cortical region targeted: while some studies reported performance benefits with motor cortex stimulation (Vitor-Costa et al., 2015), Etemadi et al. (2023) observed significant enhancements only with DLPFC stimulation, suggesting the interaction between intensity and cortical target specificity. These mixed findings should not be viewed as contradictory but rather as illustrative of the nuanced interaction between stimulation parameters. Thus, future research must explicitly evaluate these interactions systematically, considering calcium-dependent plasticity mechanisms and avoiding simplistic linear dose assumptions. This optimised approach should be considered as the preferred methodology to test the possible performance enhancing effects of tDCS.

## 3 Summary and directions for future research the optimisation of tDCS applications in exercise performance research

This review comprehensively examines the core methodological components of tDCS administration, emphasising stimulation timing, intensity, electrode size and current density, as well as electrode montage as critical variables for optimising performance improvements. The timing of stimulation relative to exercise is a crucial yet often underreported methodological variable. Offline tDCS, applied prior to exercise, is common, is widely used, yet precise reporting of the interval between stimulation cessation and exercise initiation remains sparse, impairing reproducibility and interpretability. Evidence suggests CSE typically peaks approximately 15–30 min post-stimulation; however, longer intervals or suboptimal delays could diminish or even reverse these beneficial effects. Additionally, considerable variability exists in how online tDCS (stimulation applied during exercise) modulates CSE. Further, the relationships between stimulation duration, intensity, and subsequent performance outcomes are still not clearly understood, highlighting a need for systematic dose–response investigations. Rigorous exploration of electrode montage—including electrode size and optimal current density, precise placement, and configurations—is essential to ensure accurate targeting of relevant brain regions aligned with the specific physiological and psychological demands of different exercise tasks. Wherever feasible, employing advanced techniques such as MRI-guided neuronavigation and individualised electric-field modelling could substantially enhance spatial precision and reproducibility. Additionally, directly measuring CSE or other physiological and psychological mechanisms hypothesised to mediate performance outcomes is vital to ensure valid interpretation of observed effects. Advanced methodologies, such as HD-tDCS and extracephalic montages, require rigorous empirical validation to confirm their efficacy. Lastly, Given the non-linear interactions among stimulation parameters—timing, intensity, and montage—systematic within-subject comparisons remain essential for identifying optimal and reliable tDCS protocols.

Despite methodological advancements evidence supporting the efficacy of tDCS in enhancing exercise performance remains inconclusive. This ambiguity primarily arises from inconsistent methodological practices, such as reliance on the 10–20 EEG system for electrode placement rather than more precise approaches, omission of exact reporting regarding timing between stimulation cessation and exercise initiation, and neglect of methodological optimisations identified in broader behavioural research literature. Wherever feasible, advanced targeting techniques such as MRI-guided neuronavigation should complement or replace traditional EEG-based electrode placement, combined with accurate spatial-coordinate reporting and individualised electric-field modelling to enhance reproducibility and precision. Additionally, systematic dose–response studies are needed to critically evaluate the prevalent assumption that increasing stimulation intensity or duration linearly enhances performance outcomes. Future research should also explicitly document timing decisions based on established neurophysiological rationales and systematically evaluate multiple timing conditions. Furthermore, to ensure robust interpretation of findings, studies must directly assess corticospinal excitability or other relevant physiological and psychological mechanisms hypothesised to mediate exercise-performance improvements. By incorporating these methodological improvements—precise neuronavigation where possible, systematic dose assessments, explicit timing reporting, direct measurement of the targeted mechanisms, and alignment between the specific exercise component and targeted brain region—future research will significantly enhance the understanding of tDCS effectiveness and clarify its practical utility in improving exercise performance. Table 1 summarises recommended optimised tDCS methodologies.

**TABLE 1:** Optimised tDCS parameters and recommendations for methodological standardisation in exercise performance research.

Dosage parameter	Dosage component	Current evidence summary	Recommendations for future research
Timing	Online vs. Offline tDCS	Predominantly offline application; limited exploration of online effects	Conduct direct comparisons of online and offline tDCS during various exercise tasks
	Multiple Stimulation Sessions	Preconditioning (multiple sessions) may enhance subsequent tDCS effects through metaplasticity	Investigate methods of optimising metaplasticity-related changes in CSE for the enhancement of endurance-based performance outcomes
	Stimulation Duration	Duration typically varies between 10 and 20 min, affecting after-effects and performance outcomes	Standardise stimulation durations and explore the optimal duration for specific performance tasks
Intensity	Current Intensity	Non-linear relationship observed between intensity and corticospinal excitability; inconsistent results at common intensities (1.5 mA, 2 mA)	Examine a broader range of intensities (>2 mA) to identify thresholds and potential performance improvements
Montage	Targeted Brain Regions	Mixed results from various targeted regions (motor cortex, DLPFC) due to varying outcomes measured. Methods of targeting often rely on 10/20 EEG system without precise anatomical localisation	Match targeted brain regions specifically with distinct exercise components (e.g., pain tolerance, motor coordination, affective response). Employ advanced localisation methods such as neuronavigation and MRI-guided targeting for precise stimulation placement
	High-Definition (HD-tDCS)	Improved focality and excitability, but currently limited and inconsistent evidence for exercise enhancement	Expand studies using HD-tDCS to systematically assess efficacy across diverse exercise modalities and intensities
	Extracephalic Montage	Potentially greater focality and deeper cortical stimulation; empirical validation remains limited	Conduct rigorous experimental validation of extracephalic montages to confirm their efficacy in various performance tasks
Electrode	Electrode Size	Electrode sizes vary considerably (typically 12–35 cm^2^), influencing current density and stimulation focality	Standardise electrode sizes and systematically investigate optimal sizes for various performance outcomes. Clearly report electrode specifications for better comparability across studies
Parameter Interactions	Dose Parameter Interactions	Complex, non-linear interactions between timing, intensity, and montage significantly influence performance outcomes	Conduct systematic investigations into how timing, intensity, and montage parameters interact to optimise tDCS protocols for reliable exercise performance enhancement

## 4 Concluding remarks

This critical literature review provides a detailed and integrated analysis of methods employed to optimise tDCS administration for enhancing human exercise performance. It highlights the significant scope for methodological improvement, particularly in systematically manipulating dosage parameters (timing, intensity, current density, electrode montage), documenting and exploring the relationship between stimulation and exercise cessation and initiation to enhance reproducibility, and challenging assumptions around linear dose–response relationships. Adopting more advanced methodologies such as MRI-guided neuronavigation where feasible, novel electrode montages, and systematic dose assessments are essential steps toward establishing reliable and generalisable outcomes. By systematically addressing these methodological recommendations, future research can enhance the efficacy, consistency, and practical utility of tDCS applications, ultimately maximising its potential for both scientific research and practical applications within sports contexts.
